# A case report of clonidine induced syncope: a review of central actions of an old cardiovascular drug

**DOI:** 10.1186/s40360-018-0198-1

**Published:** 2018-02-13

**Authors:** Alexander J. Sandweiss, Christopher M. Morrison, Anne Spichler, John Rozich

**Affiliations:** 1 University of Arizona, Department of Pharmacology, College of Medicine, 1501 N. Campbell Ave LSN 621, Tucson, AZ 85724 USA; 2 University of Arizona, Department of Medicine, College of Medicine, Tucson, AZ USA; 3 Southern Arizona Veterans Affairs Health Care System, Tucson, AZ USA

**Keywords:** Basic pharmacology, Clonidine, Brainstem

## Abstract

**Background:**

Clonidine is an imidazoline sympatholytic, acting on both α_2_-adrenergic and imidazoline receptors in the brainstem to induce antihypertensive and negative chronotropic effects in the vasculature and heart respectively.

**Case presentation:**

A 69-year-old gentleman with hypertension presented to the emergency department after multiple syncopal episodes over the past 12 months. Electrocardiogram demonstrated sinus bradycardia with a heart rate of 42 beats per minute. It was hypothesized that the antihypertensive agent clonidine was responsible for inducing symptomatic bradycardia. Clonidine was thus gradually tapered and then discontinued over five days restoring normal sinus rhythm rates while avoiding hypertensive rebound related to sympathetic surge. His heart rate and blood pressure remained within normal limits after the clonidine taper and subsequent adjustments to his other hypertensive medications and he was discharged.

**Conclusions:**

While clonidine has fallen out of favor for its indication as an antihypertensive, it remains a viable option for the use of opioid withdrawal, chronic pain, and smoking cessation, necessitating the appropriate clinical and pharmacological competencies for a physician to prescribe. A discussion of the clinical effects of clonidine brainstem receptor activation follows.

## Background

Cardiovascular drugs rank second to narcotics [1] as a source of adverse drug events and fatal poisonings in the U.S. Among adverse events, hypotension and dysrhythmias, including profound symptomatic bradycardia, represent significant risks for patients, especially the elderly *(and is thus on the Beers criteria for potentially inappropriate medications to use in the elderly* [2]*).* One cardiovascular drug, clonidine, is an imidazoline compound shown to have potent hypotensive actions in animals and humans [3]. Previously marketed as ‘Catapres,’ this sympatholytic drug has been clinically shown to reduce blood pressure and heart rate long before the discovery of G-protein coupled receptors such as the α_2_-adrenergic receptors were mechanistically linked to its phenotypic effect [4, 5]. While the initial reports of clonidine’s actions on the vasculature and heart provided ample evidence for its depressor and negative chronotropic actions respectively [6, 7], it remained unclear where in the body this agent exerted its pharmacological effects [8]. However, its robust hemodynamic properties including its potential for adverse outcomes including syncope [9] were evident early after its introduction into medical practice and remain a cautionary note to those continuing to employ it. Here, we present a case of clonidine adverse drug reaction and go on to discuss recent advances in the literature on clonidine pharmacology.

## Case presentation

A 69-year-old male with a past medical history of insulin dependent diabetes (A1C = 6.3%), diabetic peripheral neuropathy, and no known coronary artery disease (CAD) or prior dysrhythmias was evaluated. He had a distant history of a transient ischemic attack, long standing hypertension, and no history of seizures or prior cerebrovascular accident (CVA). He presented to the emergency department (ED) shortly after a syncopal event while in the parking lot walking from his car to a grocery store. He had three prior syncopal events in the last year requiring hospitalization. He reported feeling dizzy prior to the most recent fall. As a consequence of the fall, he struck the right side of his face against the asphalt. He then regained consciousness and was able to resume an upright posture immediately. Upon arrival in the ED he was asymptomatic as he denied chest pain, shortness of breath, abdominal pain, headache, changes in vision, speech, strength, sensation, or gait. He noted that he often felt dizzy at least twice per week and that this persisted over the last several months. His current meds included simvastatin, clopidogrel, gabapentin, insulin, lisinopril, amlodipine and clonidine. He denied tobacco/illicit drug use and endorsed occasional alcohol use. He denied over the counter medications or therapies.

**Fig. 1:**
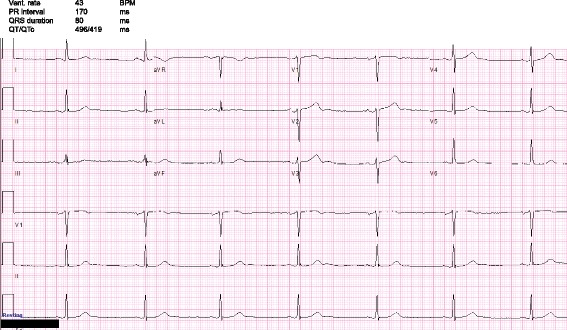
Electrocardiogram at admission demonstrates sinus bradycardia

**Fig. 2:**
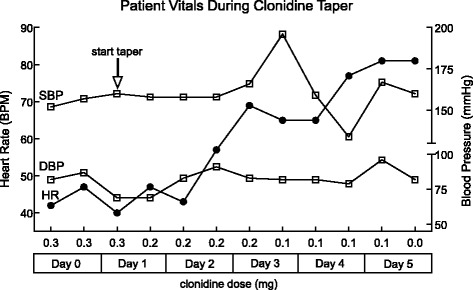
SBP, systolic blood pressure; DBP, diastolic blood pressure; HR, heart rate; BPM, beats per minute

Upon arrival, he was afebrile, pulse 42 BPM, respirations 16/min, blood pressure 164/62 *mmHg*, and O_2_ saturation was 97% on room air. His physical exam revealed an alert, oriented male with normal cognition. The remainder of his exam including a detailed neurological exam was unremarkable. Chest x-ray and head CT were normal. Blood work revealed normal electrolytes and glucose, with a negative troponin. Electrocardiogram (EKG) (Fig. 1) revealed marked sinus bradycardia. The patient was admitted and placed on telemetry for recurrent symptomatic sinus bradycardia. There were no ST or T wave or other EKG changes and the QT and QTc were normal (496 and 419 ms respectively). Further questioning revealed the patient was started on the clonidine for hypertension about 18 months prior. Due to a high degree of clinical suspicion early on in the hospital course, clonidine was discontinued from 0.3 mg BID over the course of five days to avoid severe rebound hypertension from sympathetic surge [10] (Fig. 2). His bradycardia resolved by day 2-3 of the taper and recurrent hypertension was alternatively treated. The patient was discharged with a pulse of 78 BPM and BP of 160/82 *mmHg*.

Over the course of his hospitalization, other potential etiologies were considered, including polypharmacy leading to pharmacodynamic and pharmacokinetic interactions as well as complications due to diabetes including autonomic neuropathy [11]. Diabetic autonomic neuropathy was less likely given that the cessation of clonidine relieved the bradycardia, although a synergistic effect of the drug and diabetes certainly cannot be ruled out.

## Discussion and conclusions

Clonidine is an imidazoline sympatholytic, rendering it highly efficacious as a vascular depressor and negative chronotropic agent. The patient described presented with multiple episodes of syncope thought to be most likely due to bradycardia. This is a foreseeable outcome given the mechanism of this agent, an α_2_-adrenergic receptor agonist, coupled to lowering serum noradrenaline levels [12]. While the cardiovascular phenotypic consequences of clonidine are obvious, the incidence of adverse drug reactions to clonidine has not been sufficiently investigated [13]. Our focus is the enhanced understanding of clonidine’s basic molecular pharmacology that has occurred over the past several decades.

It has long been hypothesized that clonidine acts at critical foci within the the medulla of the brainstem. Here it exerts its effects on the autonomic nervous system to attenuate sympathetic outflow [14]. The rostral ventrolateral medulla (RVLM) is a nucleus considered by many to be the site of sympathoexcitatory generation, with efferents contributing powerfully to both vasomotor and chronotropic targets [15]. Within this specific brainstem nucleus exist barosensitive neurons synchronized to the cardiac cycle [16]. Foreseeably, when the baroreceptors detect a drop in vascular pressure, the efferent catecholaminergic response is then potentiated. Clonidine and other imidazoline derivatives likely exert their effects in this region to terminate presynaptic release of norepinephrine, and it is proposed they do so via their pharmacological actions on α_2_ or the controversial imidazoline receptors [17–19]. Furthermore, the cardio-inhibitory effect of clonidine is functionally antagonized by the local application of *N-methyl-D-aspartate* receptor (NMDA) blockers, highlighting the importance of glutamate signaling in cardiovascular depression in the RVLM [20, 21].

But it is only recently that evidence has been provided that clonidine is mechanistically coupled to both *reducing sympathetic* but also *increasing parasympathetic* outflow to the cardiovascular system. The primary parasympathetic driver to the heart comes from the premotor cardioinhibitory vagal neurons in the nucleus ambiguus of the medulla, which receives input from multiple brainstem nuclei including the nucleus tractus solitarius (NTS) [22, 23]. Inhibitory GABAergic input into the nucleus ambiguus prevents activation of the parasympathetic nuclei [24, 25]. Clonidine prevents the release of GABA in the nucleus ambiguus, thus *disinhibition* of that cluster of neurons potentiates parasympathetic outflow to the heart [26].

In addition to the central actions of clonidine, there is new additional evidence of a direct peripheral tissue effect. At the level of target cardiac and vascular structures, substantial expression of α_2_-adrenergic receptors exists where clonidine prevents the vesicular release of norepinephrine, thus functionally antagonizing local myocyte β-adrenergic activation.

It was clear from its first description in the 1960s that clonidine possessed excellent sympatholytic activity, reducing blood pressure and slowing the heart. Its aforementioned negative chronotropic effects can thus predictably lead to overt bradycardia and syncope [27]. While it has fallen out of favor as a first line antihypertensive agent, new reports have shown efficacy in chronic pain [28], Tourette’s Syndrome [29], opioid withdrawal [30], and smoking cessation [31]. It is still employed for resistant hypertension, revealing that it still has an important adjunctive role. But foreseeably, its less frequent use as a hypertensive agent has resulted in clinicians becoming increasingly unfamiliar with its pharmacology and therapeutic outcomes. And finally, the collective understanding of the molecular basis for clonidine’s mechanistic impact on cardiovascular hemodynamics continues to evolve. Understanding the molecular and cellular basis of inhibiting sympathetic tone may provide a crucial link to the clinical phenotypic expression of syncope.

## Abbreviations

CAD: coronary artery disease
CVA: Cerebrovascular accident
ED: emergency department
EKG: electrocardiogram
NMDA: *N-*methyl*-D-*aspartate
NTS: nucleus tractus solitarius
RVLM: rostral ventrolateral medulla
